# *TP53* oncogenic variants as prognostic factors in individuals with glioblastoma: a systematic review and meta-analysis

**DOI:** 10.3389/fneur.2024.1490246

**Published:** 2024-12-18

**Authors:** Diego Esperante, Kena Daza Galicia, Kalu Gabriel Rivas-Cuervo, Bernardo Cacho-Díaz, Catalina Trejo-Becerril, Lucia Taja-Chayeb, Orwa Aboud, José Alberto Carlos-Escalante, Talia Wegman-Ostrosky

**Affiliations:** ^1^Combined Studies Plan in Medicine (PECEM), Universidad Nacional Autónoma de México (UNAM), Mexico City, Mexico; ^2^Faculty of Medicine, Universidad Nacional Autónoma de México (UNAM), Mexico City, Mexico; ^3^Faculty of Superior Studies (FES) Iztacala, Universidad Nacional Autónoma de México (UNAM), Mexico City, Mexico; ^4^Fundación Gimnasio Moderno, Bogota, Colombia; ^5^Neuro-Oncology Unit, Instituto Nacional de Cancerología, Mexico City, Mexico; ^6^Precision Medicine Laboratory, Sub-Direction of Research Unit, INCan, Mexico City, Mexico; ^7^Neuro-Oncology, Department of Neurology and Neurosurgery, UC Davis Comprehensive Cancer Center, Sacramento, CA, United States

**Keywords:** glioblastoma, *TP53*, brain tumors, survival, meta-analysis, prognosis

## Abstract

**Background:**

This systematic review and meta-analysis investigated the relationship between somatic *TP53* oncogenic variants and prognosis, specifically with overall survival (OS) and progression-free survival (PFS) in patients diagnosed with supratentorial glioblastoma.

**Methods:**

We included longitudinal studies and clinical trials involving a minimum of 40 adult participants diagnosed with supratentorial glioblastoma, wherein the status of *TP53* variants was assessed. We conducted searches in multiple databases. We assessed bias risk using a modified version of the Quality in Prognosis Studies tool, and the certainty of evidence was evaluated following the principles of the GRADE approach.

**Results and conclusion:**

This study encompassed 23 papers involving 2,555 patients, out of which 716 had reported oncogenic variants. *TP53* oncogenic variants were associated with a reduced likelihood of 1-year survival (OR 0.52, 95% CI 0.29–0.94). However, our analysis did not reveal any significant impact of *TP53* variants on overall survival, progression-free survival, or 2-year survival. Therefore, this comprehensive analysis demonstrates that the presence of genetic variants in *TP53* does not provide useful information for the prognosis of glioblastoma.

**Systematic review registration:**

https://www.crd.york.ac.uk/prospero/, identifier CRD42021289496.

## 1 Introduction

Glioblastoma stands as the most prevalent and aggressive primary malignant tumor of the central nervous system, representing a formidable clinical challenge (1). Despite comprehensive treatment strategies encompassing surgery and chemoradiation, patients diagnosed with glioblastoma face a daunting prognosis, marked by a median overall survival (OS) of merely 15.6 months (2).

The diagnostic landscape of central nervous system tumors underwent a significant transformation with the advent of the World Health Organization (WHO) 2016 classification of Tumors of the Central Nervous System (TCNS). This revision incorporated diagnostic molecular factors into the standard histopathological evaluation. Among these pivotal biomarkers, *IDH1/2* somatic variants are critical in confirming the diagnosis of glial tumors, including glioblastoma, and distinguishing them from lower-grade counterparts (3). Furthermore, the WHO published a new TCNS classification in 2021, focusing more on tumors’ genetic landscape. This update presents new tumor types and subtypes and includes a reclassification of specific tumors based on their genetic profile and a reviewed tumor taxonomy (4).

*TP53* variants exhibit a dual presence within glioblastoma patients’ germline and somatic lineages (5). Germline pathogenic variants in *TP53* are associated with Li-Fraumeni syndrome, a hereditary cancer predisposition syndrome (6). On the other hand, somatic *TP53* oncogenic variants manifest at a prevalence ranging from 15 to 36% among glioblastoma cases (7, 8). These variants typically occur within exons 5–8, predominantly clustering in hotspots intricately linked to the DNA-binding domain of the *TP53* protein (9).

While *TP53* oncogenic variants have earned notoriety for their adverse prognostic implications in various cancer types, including breast cancer, esophageal carcinoma, and leukemia, their precise role in dictating outcomes for glioblastoma patients remains an enigma (10, 11). Despite their relatively common occurrence, a definitive association between *TP53* status and prognosis in glioblastoma remains elusive.

Therefore, this systematic review and meta-analysis were undertaken to elucidate the intricate relationship between somatic *TP53* oncogenic variants and the prognosis of individuals grappling with glioblastoma. We define our inquiry utilizing the PICOTS framework, delineating the population, index prognostic factor, comparator prognostic factor, outcome(s), timing, and setting.

## 2 Methods

We followed the Preferred Reporting Items for Systematic Reviews and Meta-Analysis (PRISMA) Statement. We registered our systematic review in PROSPERO (registry number: CRD42021289496).

### 2.1 Eligible studies

Eligible studies: reports with publication dates after 2008, data from humans, a minimum sample size of 40 eligible individuals whose oncogenic variants status had been evaluated by DNA sequencing or PCR, longitudinal studies, and clinical trials. The minimum number of participants was 40 to avoid or minimize the risk of small-study effects and exclude case series.

Eligible participants: individuals with a pathologic confirmed diagnosis of glioblastoma, aged ≥18 years. If an article reported summary data from a mixed pediatric and adult cohort, we pondered its inclusion only if the pediatric component was <10%. Tumors from eligible participants had to be localized in the intracranial supratentorial compartment of the brain. If an article provided specific information about anatomical localization, it was considered for inclusion only if the aggregated fraction of infratentorial and spinal tumors was less than 10%. We generally chose a 10% margin for pediatric patients and infratentorial tumors to avoid discarding studies with more prominent participants. Furthermore, individual patient data (IPD) were reported in most cases. Studies that included participants that did not meet all the inclusion criteria were included only if an out-lined sub-analysis of eligible participants was performed or if individual participant data (IPD) was reported.

In this work, we avoided the use of the word “mutation”; while this term is widely used to describe changes in the nucleotide sequence, it is no longer recommended; instead, we used the term “genetic variant” as proposed by the Joint recommendations of Clinical Genome Resource (ClinGen), Cancer Genomics Consortium (CGC), and Variant Interpretation for Cancer Consortium (VICC). Further classification of somatic genetic variants in the context of cancer is possible with the following five categories: oncogenic (O), likely oncogenic (LO), a variant of uncertain significance (VUS), likely benign, and benign (12).

### 2.2 Search strategy and screening process

Our systematic search, conducted until October 1st, 2022, encompassed several critical databases: PubMed, Web of Science, Scopus, Biblioteca Virtual en Salud (a search engine aggregating 53 databases, excluding MEDLINE), and OpenGrey. We confined our search to English, Spanish, or Portuguese manuscripts.

To ensure rigor and impartiality, two authors conducted the initial screening of articles independently, employing the search algorithm detailed in Supplementary material 1. When disagreements arose concerning the inclusion or exclusion of specific articles, a constructive dialogue between the two investigators ensued. In cases where a unanimous decision remained elusive, a third author intervened as a tiebreaker to facilitate consensus.

### 2.3 Data extraction

Data from the included studies were extracted and collected in spreadsheets individually by five authors: DE, KDG, KGRC, JACE, and BCD—another corroborated data extracted by one author. Time-to-event data comparing individuals grouped by *TP53* for overall survival (OS) and progression-free survival (PFS) outcomes were extracted as hazard ratios (HRs). In contrast, survival data presented as dichotomous (i.e., 1-year or 2-year survival) was recorded as odd ratios (ORs). Adjusted effect measurements were preferred over unadjusted, particularly when adjusting included the following molecular and clinical variables: *IDH1* or *IDH2* oncogenic variant status, age, sex, the extent of resection, chemotherapy, radiotherapy, and functional status. The 95% confidence intervals (95% CI) and *p* values were also extracted for all effect measurements. If the information of interest was not directly provided in the article but IPD was available, we calculated HRs and ORs, adjusting for relevant variables reported in the IPD. Without clearly reported HRs or individual patients’ data, HRs were calculated from Kaplan–Meier curves if image resolution was adequate. Heterogeneity was evaluated with the Cochran Q test and the Higgins I^2^ statistics.

### 2.4 Assessment of risk of bias

The risk of bias was assessed with a modified version of the Quality in Prognosis Studies (QUIPS) tool (Supplementary material 2) (13). This modified QUIPS tool was based on the version used by McAleenan et al. (14).

### 2.5 Data synthesis

We used a random effects model and evaluated the risk of publication bias in each meta-analysis if it included ten or more studies. We expected to synthesize time-to-event survival data through HRs; therefore, we used a modified version of Peter’s test, proposed by Debray, Moons, and Riley (15). For meta-analyses pooling odds ratio, we used Egger’s test. Contour-enhanced funnel plots were created with the “funnel” command from the package metaphor for R (v. 3.8-1). Finally, the certainty of evidence evaluation was carried out according to the principles proposed by the GRADE approach (16).

## 3 Results

### 3.1 Study selection

**Figure 1:**
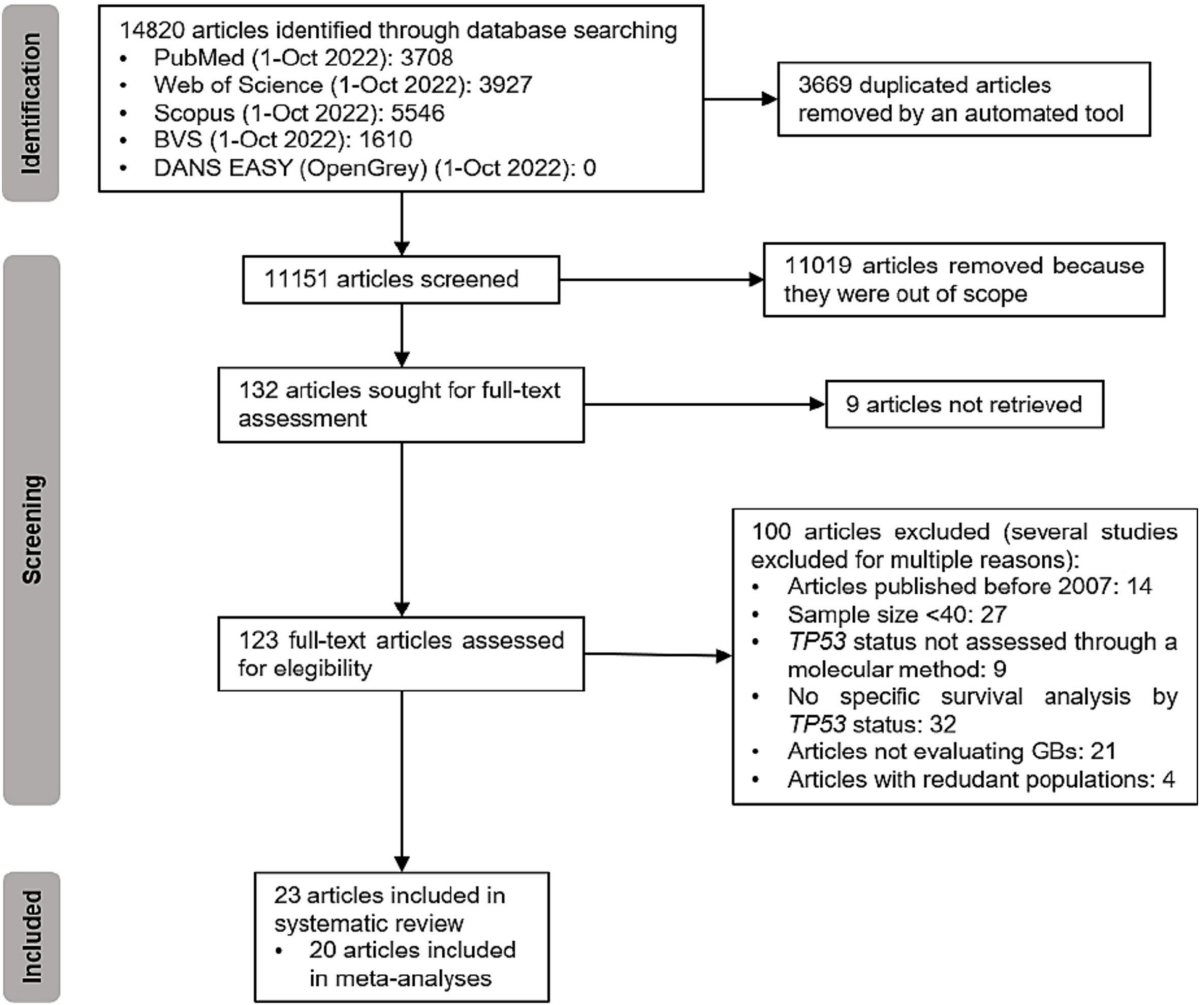
PRISMA flowchart for the inclusion of articles in this review.

Our initial database search yielded a substantial pool of 14,820 manuscripts. After rigorous screening and assessment, a total of 23 manuscripts were deemed eligible for inclusion in our analysis, as illustrated in Figure 1 and Table 1 (7, 8, 17–37). Notably, it’s worth mentioning that the publication authored by Yang et al. (37) encompassed two distinct studies. It’s also important to note that three of the initially considered reports, specifically those authored by Felsberg et al. (7), Jesionek-Kupnicka et al. (23), and Motomura et al. (26), lacked the essential quantitative data required for inclusion in the quantitative synthesis. Therefore, they were only summarized in the Systematic Review. The studies included in our systematic review are summarized in Table 2.

**Table 1:** PICOTS question of this systematic review.

Population	Adults diagnosed with supratentorial GB
Index prognostic factor	Presence of somatic *TP53* genetic variants detected in tumoral tissue with at least one of the following methods: PCR, high-throughput DNA sequencing, Sanger sequencing, or Maxam-Gilbert sequencing
Comparator index factor	Not applicable
Outcomes	Overall survival (time-to-event data, in months), progression-free survival (time-to-event data, in months), 1-year survival (dichotomous outcome, rate), 2-year survival (dichotomous outcome, rate), 5-year survival (dichotomous outcome, rate).
Time	Any follow-up period
Setting	Any setting

**Table 2:** Studies included in the systematic review.

References	Country	Median age at diagnosis	Method to detect *TP53* variants	Number of patients (*TP53 oncogenetic variant*)	Outcomes evaluated
Clark et al. (19)	USA	60	PCR-SSCP (exons 5–8)	48 (11)	OS, 1y-mortality
Parsons et al. (27)	USA	52 *IDH*-wt: 55.5	WES	87 (25) *IDH*-wt: 76 (25)	OS, 1y, 2y
Felsberg et al. (7)	Germany	56	PCR-SSCP (exons 4–10)	65 (13)	OS
Weller et al. (34)	Germany	60.1	PCR-SSCP, then Sanger sequencing (exons 5–8)	292 (45)	OS, PFS
Benito et al. (18)	Spain	53	Sanger sequencing (exons 5–8)	45 (8)	OS, 1y, 2y
Motomura et al. (26)	Japan	55	Sanger sequencing (exons 5–8)	68 (23)	OS
Hartmann et al. (21)	Germany	Not reported for the whole cohort	PCR-SSCP, then Sanger sequencing	344 (55)	5y^b^
Jesoniek-Kupnicka et al. (22)	Poland	61 *IDH*-wt: 60	Sanger sequencing (exons 5–8)	41 (11) *IDH*-wt: 40 (10)	OS, 1y, 2y
Stancheva et al. (30)	Bulgaria	56	Sanger sequencing (exons 5–8)	106 (37)	OS
Tabone et al. (32)	Australia	63.3	Targeted sequencing (*TP53* regions: NR)	*IDH*-wt: 43 (11)	1y, 2y
Wang et al. (33)	China	49.7	PCR-SSCP, then Sanger sequencing (exons 4–8)	68 (24)	OS, 1y, 2y
Sim et al. (29)	South Korea	52.8^a^	WES	75 (25)	OS
Jesoniek-Kupnicka et al. (23)	Poland	63	Sanger sequencing (exons 5–8)	49 (12)	OS
Liu et al. (24)	USA	*IDH* wt: 62	WES	*IDH*-wt: 138 (32)	OS
McNulty et al. (25)	USA	59 *IDH* wt: 61	Targeted sequencing (all exons)	61 (21) *IDH*-wt: 54 (16)	1y, 2y
Yang et al. (36)	Korea	*IDH*-wt: 57	WES, Targeted sequencing (all exons)	*IDH*-wt: 43 (13)	OS, PFS
Qin et al. (28)	USA^d^	57	WES	149 (48)	OS
Stasik et al. (31)	Germany	*IDH*-wt: 62	Targeted sequencing (all exons)	*IDH*-wt: 55 (12)	OS, 1y, 2y
Dono et al. (20)	USA	*IDH*-wt: 61	Targeted sequencing (all exons)	*IDH*-wt: 282 (85)	OS, PFS
Wong et al. (35)	China	*IDH*-O^e^: 38	Targeted sequencing (all exons)	*IDH*-O: 53 (30)	OS
Amer et al. (17)	USA	59	Targeted sequencing (all exons)	41 (30)	OS, PFS
Pandey et al. (8)	USA	58-59^f^	Targeted sequencing (whole gene)	109 (40)	OS, PFS
Yang et al. (37) (Discovery cohort)	USA	62.2 *IDH*-wt: NR	Targeted sequencing (all exons)	185 (68) *IDH*-wt: NR	OS, PFS
Yang et al. (37) (Validation cohort)	USA	NR	Targeted sequencing (all exons)	108 (42) *IDH*-wt: NR	OS, PFS

a Only mean was reported.

b The outcome of 5-year survival was contemplated in the protocol, but only one study provided enough information.

c The analyzed cohort was TCGA, *IDH*-wt patients only.

d The analyzed cohort was TCGA, all patients.

e This was the only study analyzing enough *IDH*-mut patients.

f Ages were reported separately for control and treatment groups.

IDH, isocitrate dehydrogenase; NR, not reported; O, oncogenic variant (i.e., IDH-O); mt, mutation; OS, Overall survival; PFS, Progression-free survival; TP53, p53 gene; wt, wildtype; PFS, Progression-free survival; TP53, p53 gene; wt, wildtype.

### 3.2 The impact of *TP53* oncogenic variants on OS

**Figure 2:**
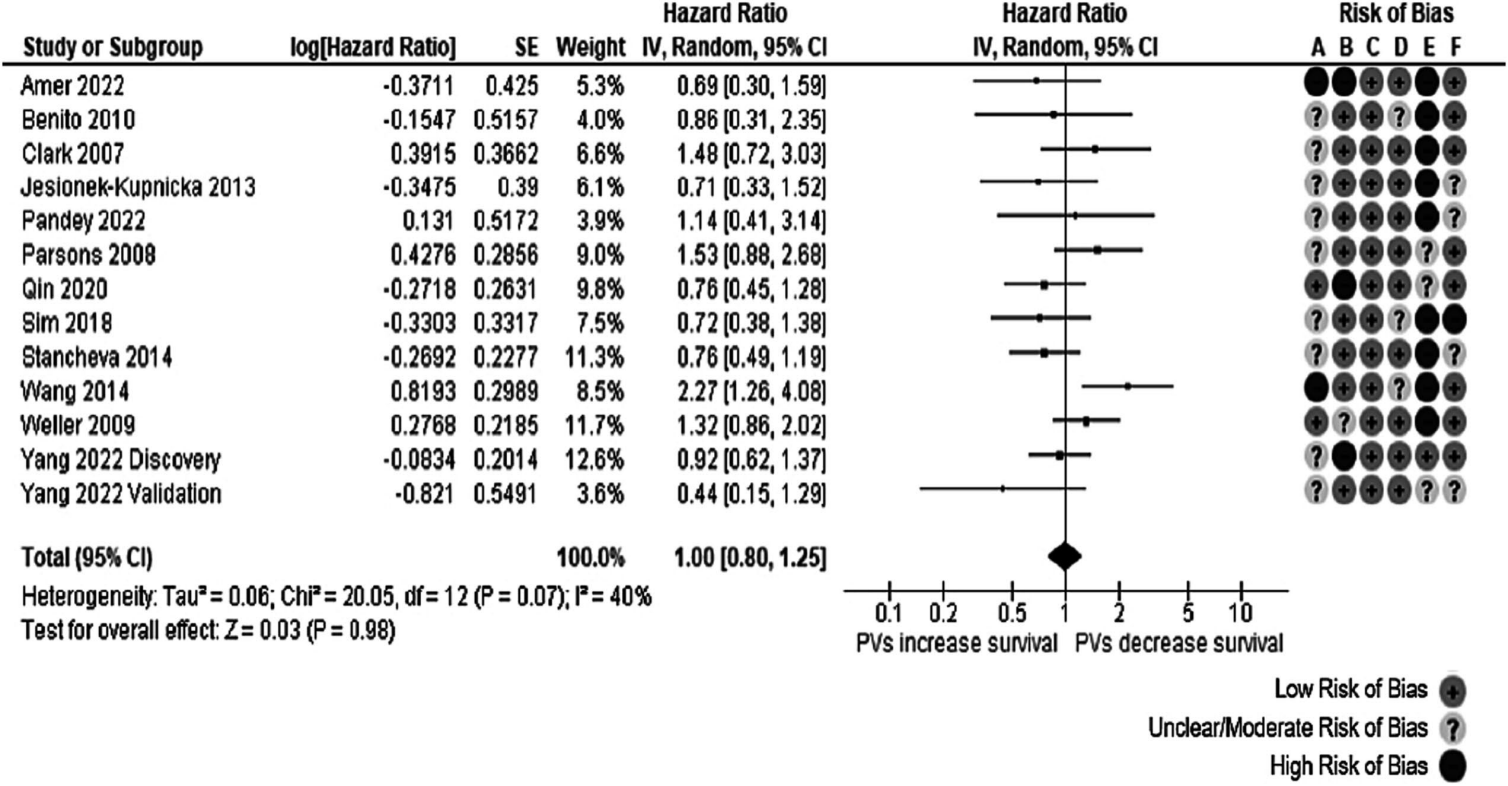
Forest plot of the meta-analysis evaluating the impact of *TP53* oncogenic variants on OS, with a summary of the risk of bias assessment through the QUIPS tool. Notice that all studies had at least one domain of the QUIPS tool rated with unclear, moderate, or high risk of bias, particularly the study confounding domain, which was only considered low risk in (36) discovery cohort.

Fifteen reports comprising 16 studies evaluated the effect of *TP53* oncogenic variants on OS, and only 13 (14 studies) provided enough quantitative data for the meta-analysis. The 14 studies reported 1,306 participants. After quantitative pooling, the occurrence of *TP53* variants was not significantly associated with OS (HR: 1.00, 95% CI: 0.76–1.19, *p* = 0.98), as observed in Figure 2. There was evidence of moderate heterogeneity in the meta-analysis (Cochran’s Q test *p*-value: 0.07), with an I^2^ of 40%. The heterogeneity found in the meta-analysis came from the studies reported by Wang et al. (33) and Parsons et al. (27). When both studies were removed from the analysis, we calculated an I^2^ of 0%. One of the possible explanations is that the patients reported in these two studies were the youngest among all the reviewed manuscripts. In addition, the study by Wang et al. (33) was designed as a clinical trial.

A *post hoc* subgroup analysis separated reports into those with selected exons (exons 4 or 5 through 8) and those where all exons were sequenced. This subgroup analysis failed to explain the heterogeneity found; the I^2^ in the “selected exons” subgroup was 55%, and in the “all exons” subgroup, the I^2^ was 10% (Supplementary Figure S1). No gross asymmetry was observed in the funnel plot (Supplementary Figure S2).

**Figure 3:**
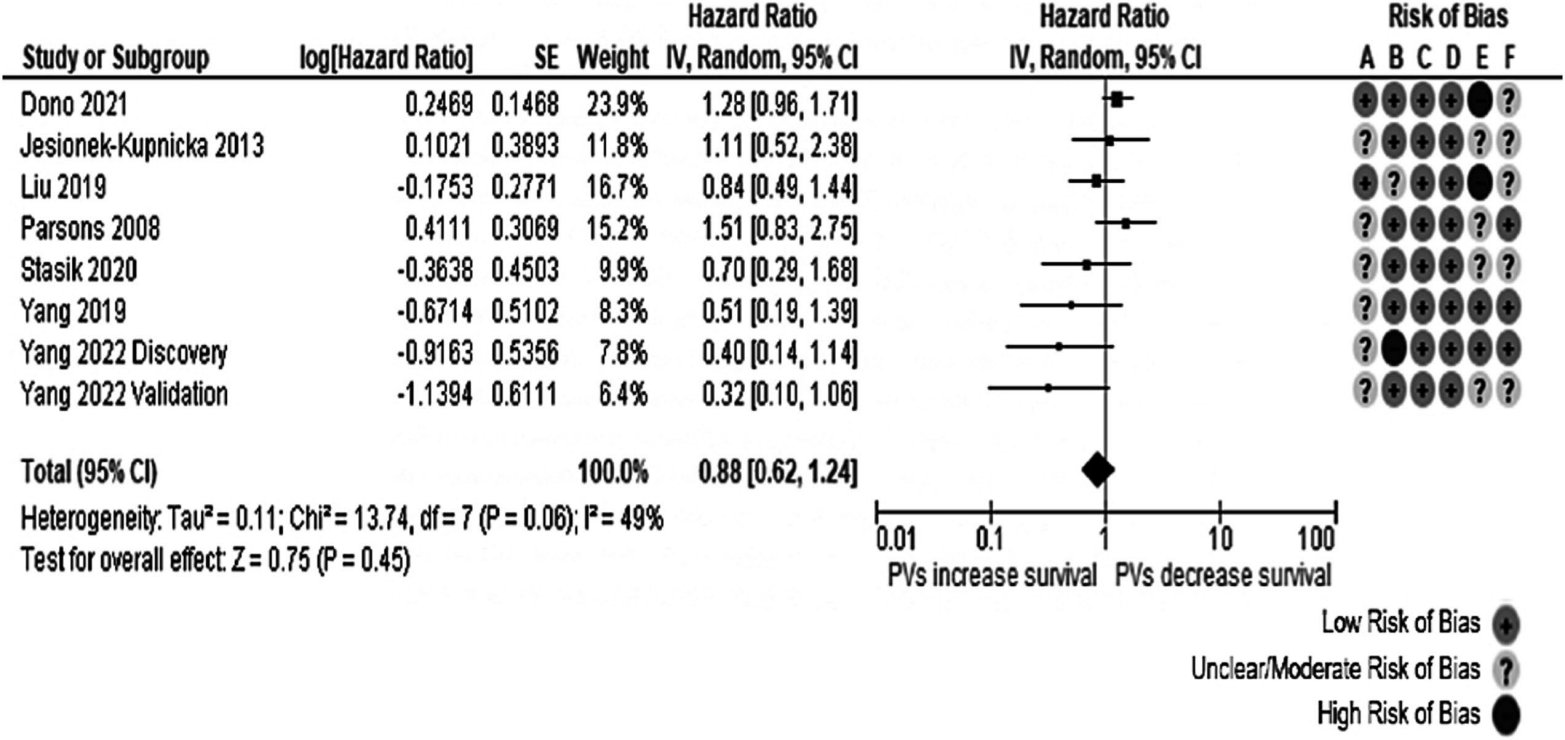
Forest plot of the meta-analysis evaluating the impact of *TP53* oncogenic variants on OS in individuals with *IDH*-wt glioblastomas, with a summary of the risk of bias assessment through the QUIPS tool. All studies had suboptimal ratings according to the QUIPS tool. Risk of Bias domains: A, Study participation; B, Study attrition; C, Prognostic factor measurement; D, Outcome measurement; E, Study confounding; F, Statistical analysis and reporting. 95% CI, 95% Confidence interval; HR, Hazard ratio; IV, Inverse variance; SE, Standard error.

We performed an additional analysis to evaluate the OS in a subgroup of patients with *IDH*-wt GB (Figure 3). The information from 8 studies with complete details (*n* = 1,125) found no effect on OS (HR: 0.96, 95% CI: 0.71–1.32, *p* = 0.82). Significant heterogeneity was uncovered for a Cochran’s Q test *p*-value of 0.06 and an I^2^ of 49%. Subgroup analyses for age or sex could not be accomplished due to insufficient information. Most of the heterogeneity in this meta-analysis came from the validation cohort (36) reported. A significant asymmetry was observed in the contour-enhanced funnel plot from this meta-analysis; all the studies fell in the white (non-significant) region, similar to where their missing counterparts would have been plotted, without mainly modifying the total effect size (Supplementary Figure S3).

### 3.3 The impact of *TP53* oncogenic variants on PFS

**Figure 4:**
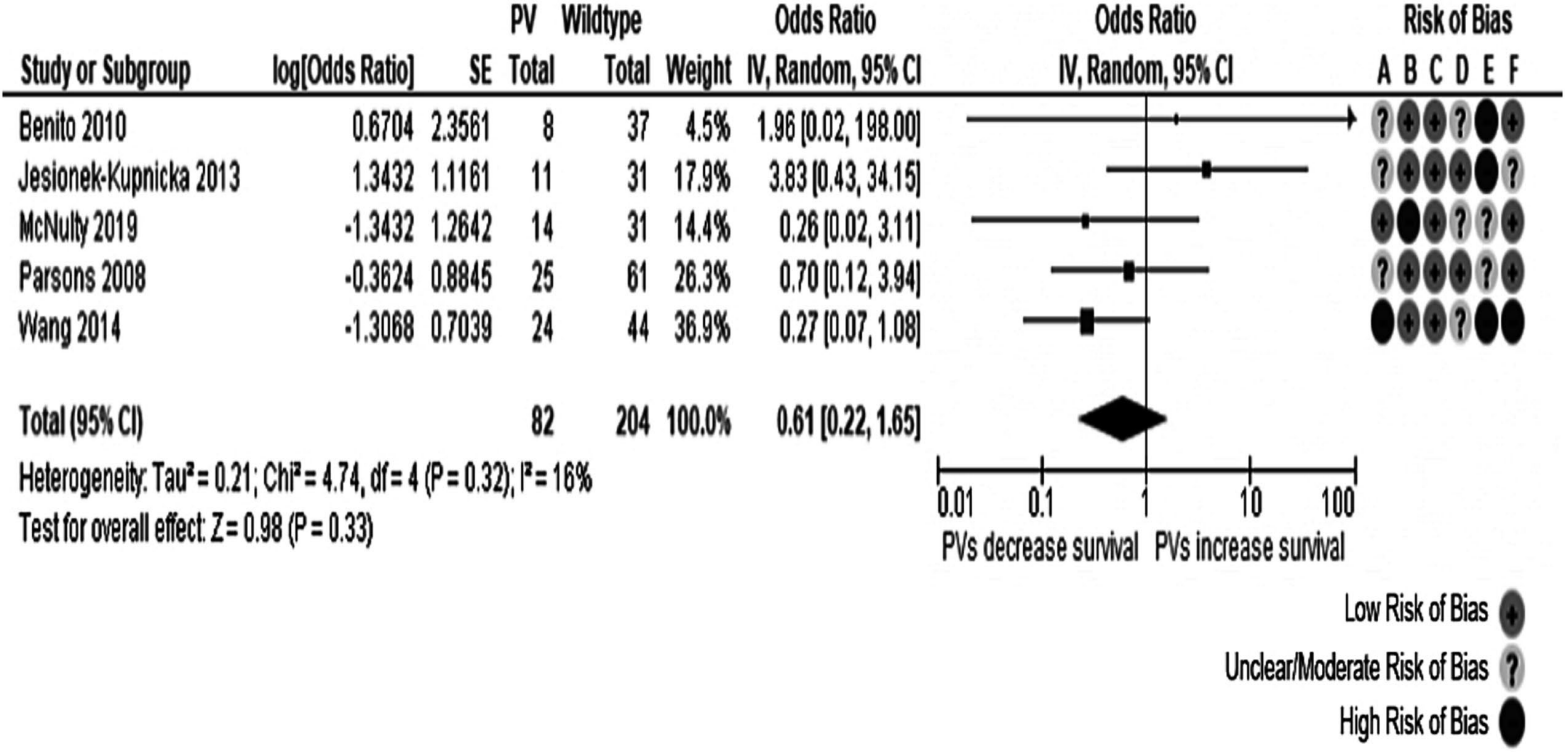
Forest plot of the meta-analysis evaluating the impact of *TP53* O/LO genetic variants on PFS in all patients with GB, with a summary of the risk of bias assessment through the QUIPS tool. All studies had suboptimal ratings in at least one domain according to the QUIPS tool. Risk of Bias domains: A, Study participation; B, Study attrition; C, Prognostic factor measurement; D, Outcome measurement; E, Study confounding; F, Statistical analysis and reporting. 95% CI, 95% Confidence interval; HR, Hazard ratio; IV, Inverse variance; SE, Standard error.

Five studies (four reports) reporting the information on 690 patients were considered to measure the association between *TP53* and PFS. The overall effect was insignificant (HR: 0.90, 95% CI: 0.79–1.39, *p* = 0.55), as observed in Figure 4. Moderate heterogeneity (Cochran’s Q test *p*-value: 0.20, *I*^2^ = 34%) was found. Removing the studies published by Amer et al. (17) and the validation cohort from Yang et al. (37), the I^2^ value was <20%. Notably, the population in the Amer et al. (17) study reported only gliosarcoma, an uncommon GB variant.

**Figure 5:**
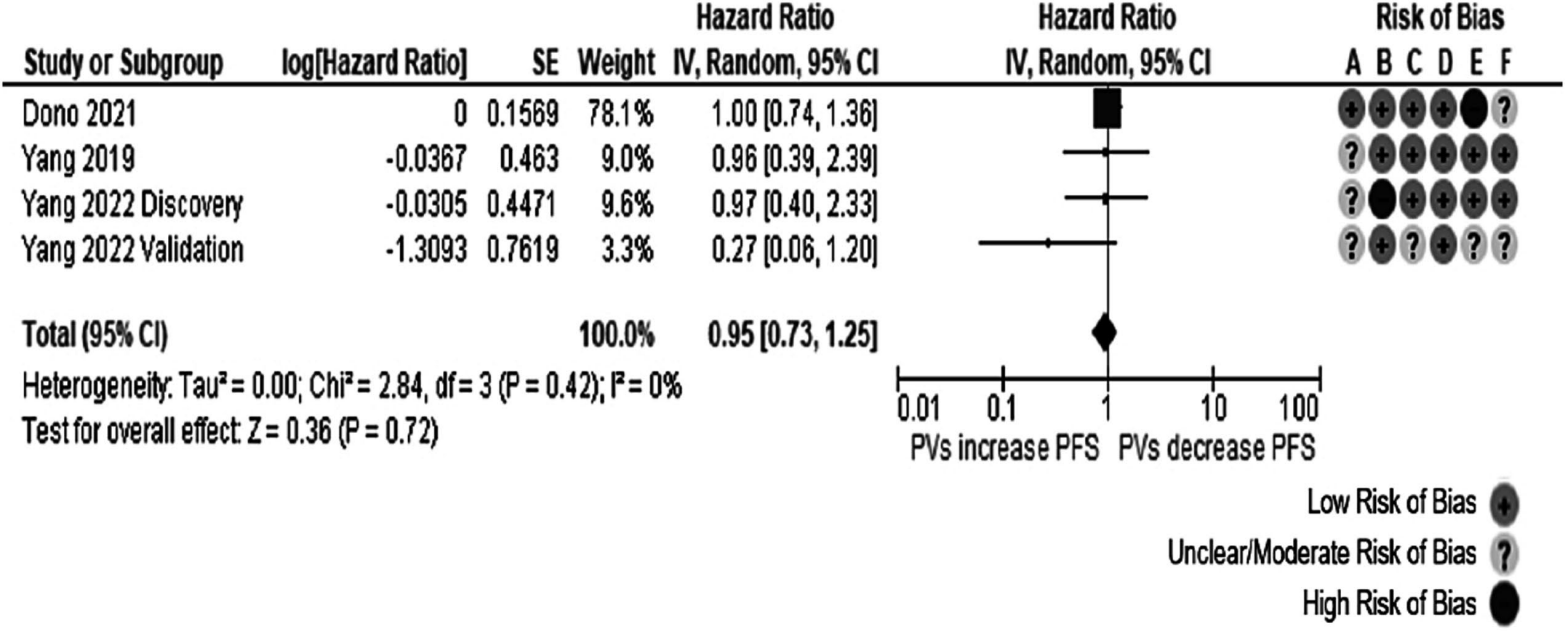
Forest plot of the meta-analysis evaluating the impact of *TP53* oncogenic variants on PFS in patients with *IDH*-wt glioblastomas, with a summary of the risk of bias assessment. The pooled effect was not statistically significant. All studies had suboptimal ratings in at least one domain according to the QUIPS tool. Risk of Bias domains: A, Study participation; B, Study attrition; C, Prognostic factor measurement; D, Outcome measurement; E, Study confounding; F, Statistical analysis and reporting. 95% CI, 95% Confidence interval; HR, Hazard ratio; IV, Inverse variance; SE, Standard error.

In the meta-analysis carried out in individuals with *IDH*-wt tumors, described in Figure 5, four studies from three different manuscripts were pooled, reporting information from 592 patients. The total effect was non-significant (HR: 0.95, 95% CI: 0.73–1.25, *p*-value: 0.72). No evidence of heterogeneity was present (Cochran’s Q test *p*-value: 0.42, *I*^2^ = 0%). In the same way, as other meta-analyses in the review, all studies had domains not rated as presenting with a low risk of bias. The Yang et al. (37) validation cohort was responsible for the funnel plot’s notorious asymmetry in both meta-analyses. Further exploration of the reasons behind the imprecision in this study was impossible due to inadequate reporting of the recruitment process and the clinical and demographical characteristics of the cohort.

### 3.4 *TP53* prognostic effect on dichotomous outcomes

**Figure 6:**
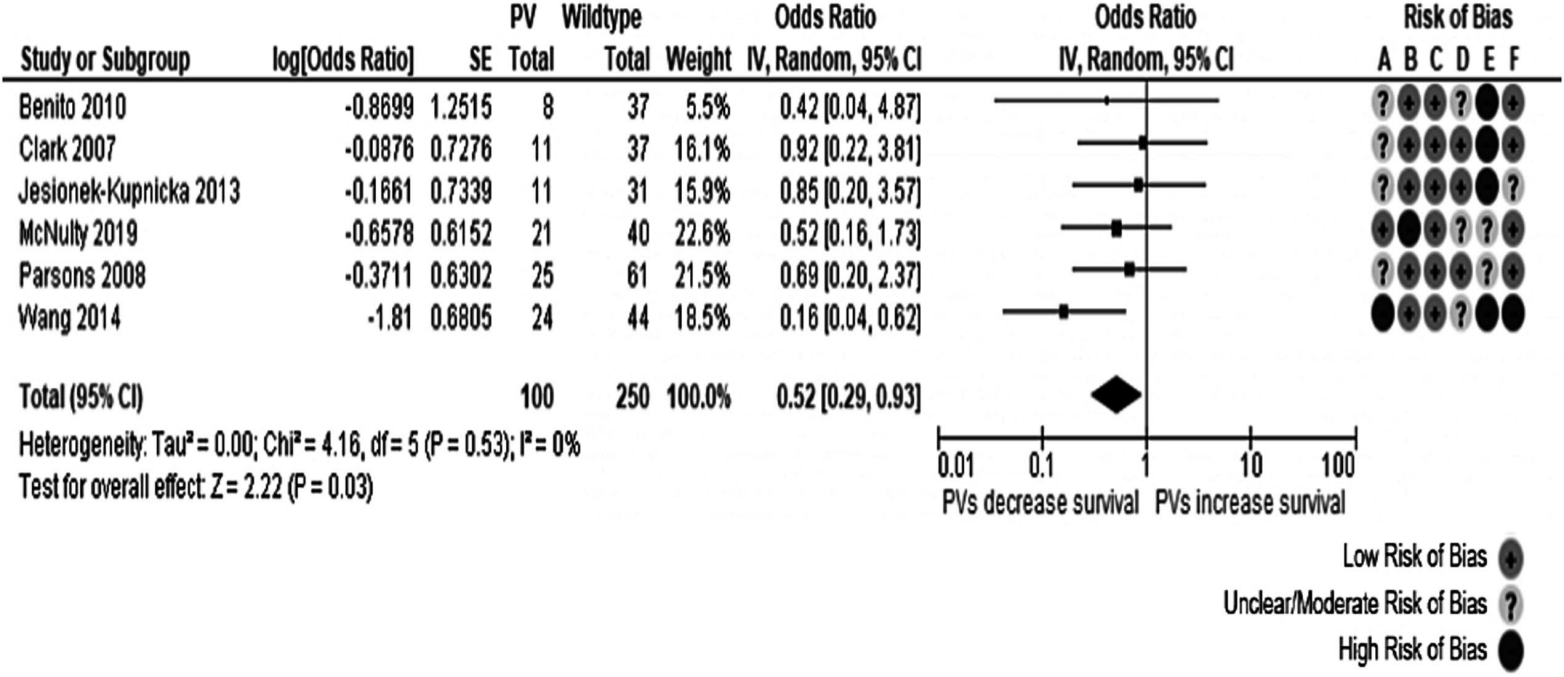
Forest plot of the meta-analysis evaluating the impact of *TP53* oncogenic variants on 1-year survival in all patients with glioblastoma, with a summary of the risk of bias assessment. Overall effect size showed a decreased 1-year survival in individuals with *TP53* genetic variants. Risk of Bias domains: A, Study participation; B, Study attrition; C, Prognostic factor measurement; D, Outcome measurement; E, Study confounding; F, Statistical analysis and reporting. 95% CI, 95% Confidence interval; HR, Hazard ratio; IV, Inverse variance; SE, Standard error.

Four meta-analyses evaluating the influence of *TP53* oncogenic variants on dichotomous outcomes for 1-year survival and 2-year survival were done. Each of them analyzed separately all glioblastoma patients and those with *IDH*-wt tumors. Six studies were pooled with information from 350 patients. *TP53* somatic oncogenic variants were associated with a lower possibility of 1-year survival in all patients with GB (OR: 0.52, 95% CI: 0.29–0.94, *p*-value: 0.03). This meta-analysis found no heterogeneity (Cochran’s Q test *p*-value: 0.44, *I*^2^ = 0%). However, all the included studies had two or three domains affected by moderate/unclear or high risk of bias, particularly the study confounding domain, as it was not rated with a low risk of bias. The corresponding forest plot is represented in Figure 6. In the contour-enhanced funnel plot, there was asymmetry in one study in the plot’s *p* < 0.01 area (gray-shaded). In contrast, the remaining four studies fell in the white area, suggesting a low likelihood of publication bias.

No significant effect was observed for any of the other meta-analyses. The meta-analyses for the 1-year survival among individuals with *IDH*-wt tumors and for the 2-year survival among all individuals with glioblastoma showed a trend toward increased mortality in individuals with oncogenic variants of *TP53* without statistical significance. Details about the three remaining meta-analyses are presented in Table 3, and their forest plots can be found in Supplementary Figures S1–S4. Asymmetries in the 1-year survival of patients with *IDH*-wt glioblastoma and the 2-year survival of all patients funnel plots were caused by Tabone et al. (32) and Wang et al. (33), respectively, with both having several domains in their risk of bias assessment rated as having unclear/moderate or high risk of bias.

**Table 3:** Results of the meta-analyses evaluating dichotomous outcomes.

		Overall effect		Heterogeneity	
Meta-analysis	Participants	OR (95% CI)	*p*-value	*I*^2^	*p*-value
1-year survival, patients with *IDH*-wildtype tumors	257	0.88 (0.46–1.70)	0.71	7%	0.37
2-year survival, all patients	286	0.61 (0.22–1.65)	0.33	16%	0.32
2-year survival, patients with *IDH*-wildtype tumors	243	1.08 (0.40–2.91)	0.88	0%	0.92

### 3.5 Summary of findings

Table 4 presents the outline of the results from our systematic review.

**Table 4:** Summary of results.

Outcome and population	Studies	Participants	HR/OR (95% CI)	*p*-value	Certainty of evidence	Reason for downvoting
OS, all GB patients	13	1,306	1.00 (0.76–1.19)	0.98	Moderate	Imprecision
OS, *IDH*-wt GB patients	8	1,125	0.96 (0.71–1.32)	0.82	Moderate	Imprecision
PFS, all GB patients	5	690	0.90 (0.79–1.35)	0.55	Moderate	Imprecision
PFS, *IDH*-wt GB patients	4	148	0.95 (0.73–1.25)	0.72	Moderate	Imprecision
1-year survival, all GB patients	6	350	0.52 (0.29–0.94)	0.03	Low	Risk of bias Indirectness
1-year survival, *IDH*-wt patients	5	257	0.88 (0.46–1.70)	0.71	High	None
2-year survival, all GB patients	5	286	0.61 (0.22–1.65)	0.33	Low	Imprecision Indirectness
2-year survival, *IDH*-wt patients	5	243	1.08 (0.40–2.91)	0.88	High	None

## 4 Discussion

In this systematic review and meta-analysis, we analyzed the impact of somatic *TP53* oncogenic variants on OS, PFS, and 1-year or 2-year survival in patients with glioblastoma. The presence of *TP53* oncogenic variants was associated with a lower 1-year survival possibility (OR: 0.52, 95% CI: 0.29–0.94) but was not related to other outcome measures, namely OS, 2-year mortality, and PFS. However, due to the nature of the meta-analysis and the studies included, it remains essential to question the integrity of the effect seen at the 1-year mark.

We performed four meta-analyses with data obtained from longitudinal studies and clinical trials reporting a minimum of 40 adult participants diagnosed with supratentorial glioblastoma, including cases of *IDH*-wt glioblastoma, which is a common finding (1). All included reports were from individuals of European or East Asian descent, while individuals of other ethnic backgrounds were underrepresented, which could bias the study. We selected only reports that included the status of somatic *TP53* oncogenic variants. However, future studies on *TP53* variants should ideally include comparison between different variants, e.g., loss versus gain of function; DNA-binding domain (exons 5–11) versus transactivation domain of the p53 protein; as well as modifier genes and/or interaction with other genes, among other considerations.

Considerable heterogeneity was observed in several of the meta-analyses performed for this review, and subgroup analysis was impossible due to incomplete clinical and demographic characteristics reporting. The heterogeneity might be attributed to three main factors. First, there was insufficient statistical adjustment since only two reports in all the systematic reviews were rated with a low risk of bias in the study confounding domain. Second, differences in medical treatments may be an important source of heterogeneity, but it was not possible to record them since only a few studies described pharmacological or radiotherapeutic regimens. Third, population differences were exemplified by the disappearance of heterogeneity after eliminating studies with younger patients from the OS meta-analysis in all individuals with glioblastoma. Other reports with an important difference in their study population were those of Amer et al. (17) and Yang et al. (37), who only included patients with gliosarcoma. The heterogeneity and asymmetry likely observed in the funnel and forest plots that included these studies derive, at least in part, from the inclusion of gliosarcoma.

The *TP53* gene is the most mutated in human cancers. Its protein, p53, acts as a transcription factor that regulates critical processes such as cell cycle control, senescence, apoptosis, DNA repair, and genomic stability, thereby protecting against cancer development. Additionally, p53 influences cellular metabolism, immune responses, ferroptosis, autophagy, and the tumor microenvironment. The activity of p53 is tightly regulated by various proteins, most notably MDM2 and MDMX. In turn, p53 directly interacts with or modulates the expression of multiple genes involved in these pathways, such as XPC, GADD45, CDKN1A, Cyclin B, Bax, Bak, Fas/FasL, SCO2, and G6PDH, among others. However, genetic variants in *TP53* not only impair these protective roles but can also endow mutant p53 with oncogenic properties, driving cancer progression, metastasis, and resistance to therapies. Given the complexity of *TP53* interactions and actions, it is necessary that future research should include not only the study of *TP53* but also its multiple interactions (38, 39). *TP53* variants can be classified based on their functional effects: loss of function (LOF), resulting in haploinsufficiency; gain of function (GOF); and a dominant-negative effect (DNE), which impairs transactivation (6). Specific *TP53* variants have been shown to influence treatment responses and survival outcomes. For example, mutations at codon 273 in astrocytoma are associated with significantly longer overall survival (OS) and increased chemosensitivity compared to wild-type *TP53*, whereas in oligodendroglioma, *TP53* mutations correlate with shorter OS (40). Additionally, *in vitro* studies have demonstrated that GOF mutations at codons 237 and 273 are linked to resistance to temozolomide treatment (41). Mutated TP53 protein, particularly within the DNA-binding domain, often forms aggregates with other proteins, including wild-type p53, leading to its inactivation. These aggregates further exacerbate the loss of normal tumor-suppressor activity (42). TP53 variants not only cause misfolding of the protein but also promote the formation of biomolecular condensates and aggregates with amyloid-like properties, particularly in the form of amyloid oligomers located in the nucleus, participating in cancer progression through loss-of-function, negative dominance, and gain-of-function pathways (43). Studies have shown the presence of amyloid oligomers of TP53 in various tumor tissues. Notably, glioblastoma cells resistant to TMZ chemotherapy exhibited significantly higher levels of these amyloid oligomers of mutated TP53 compared to glioblastoma cells with wild-type TP53 or hotspot TP53 mutations not associated with chemoresistance (44). This finding suggests a role for amyloid-like TP53 oligomers in the chemoresistance phenotype of malignant and invasive brain tumors. To study the mechanism by which mutated TP53 forms these amyloid-like aggregates, Petronilho et al. demonstrated that during the aggregation process, the DNA binding domain of TP53 undergoes phase separation before aggregation. Moreover, mutant proteins such as p.Met237Ile and p.Arg249Ser, undergo solid phase transition faster than the WT protein (45).

Importantly, this knowledge about the formation of aggregates by mutated TP53 has enabled the development of therapeutic strategies targeting the phase transitions to solid-like, amorphous, and amyloid-like states (43) or the phase separation process of TP53 (45). While our study aimed to correlate the presence of TP53 variants with prognosis, it is now clear that future research should also consider the influence of TP53 mutations on treatment response.

Several *in vitro* studies have also investigated targeting *TP53* and *MDM2* in glioblastoma, emphasizing the necessity of wild-type *TP53* for therapeutic efficacy, as certain *TP53* variants may accelerate tumor progression or alter treatment responses. There is no strong evidence linking *TP53* mutational status directly to standard therapy response in glioblastoma (46, 47).

Despite these insights, our review could not perform a meta-analysis of the impact of *TP53* variants on survival or treatment response due to insufficient data on chemotherapy protocols and the lack of individual patient data. As a result, there is currently no robust evidence to definitively establish the clinical relevance of these variants in this context.

The limitations include that some studies presented underpowered multivariate analyses, resulting in wide confidence intervals. According to the QUIPS tool, most studies had at least one domain with a suboptimal rating of risk of bias. Also, we could not evaluate the effect of *TP53* oncogenic variants in patients with *IDH*-mutant tumors due to the low number of patients reported. Another significant limitation is that this is an analysis of the impact of only *TP53* without the interaction of this gene with other genes since few studies reported this gene–gene interaction. Despite these shortcomings, the findings of the present systematic review were consistent with the selected population and the measurements done.

A systematic review has certain limitations, particularly the lack of control over key aspects of the included studies, such as the number of individuals analyzed, the data reported in each manuscript, and the follow-up periods. In the case of glioblastoma patients with a median survival of approximately 15 months, studies with follow-up periods exceeding 2 years are rare. Regarding sample size, we carefully selected studies that included a minimum of 40 patients, and although different techniques were used in the selected papers to identify TP53 gene variants (PCR-SSCP, Sanger sequencing, NGS, and WES), each of these methods is widely accepted and validated for this purpose due to their high sensitivity and specificity. Furthermore, we found a 28% prevalence of TP53 variants (716 out of 2,555 individuals), which is consistent with rates reported for glioblastoma (46). Concerning treatment regimens, glioblastoma, being relatively rare and associated with limited life expectancy, has few well-established protocols, resulting in variability in therapeutic approaches based on individual patient factors. The standard treatment remains the STUPP protocol, although alternative regimens explored in some countries have not significantly impacted survival, as demonstrated by our group (48). Another limitation of our study is the low prevalence of the disease, which results in a limited number of published cases. However, using meta-analysis allowed us to achieve statistical significance by weighing the articles based on the number of cases, and while a larger number of cases could provide greater statistical power, the current analysis remains robust.

The results gathered in this systematic review and its meta-analysis are evidence for solving the controversy surrounding the prognostic implication of *TP53* genetic variants; for now, the evidence does not support the routinary use of *TP53* sequencing as a marker of GB prognosis. In particular, this is important for low-income countries, where efforts should focus on the identification of markers that have demonstrated clinical utility for diagnosis, prognosis and/or choice of treatment, such as the identification of genetic variants in *IDH1/2* or MGMT promoter methylation should be a priority.

## 5 Conclusion

Glioblastoma is a highly aggressive disease with a survival rate of about 15 months, making improvements in diagnosis critical. In this systematic review and meta-analysis, TP53 oncogenic somatic variants were associated with decreased 1-year survival rates in glioblastoma patients; however, no significant correlations were found with overall survival, 2-year mortality, or progression-free survival. Based on current evidence, TP53 sequencing does not appear to be routinely necessary for glioblastoma prognosis. This information is especially relevant for institutions and countries with limited resources. Therefore, this comprehensive analysis demonstrates that the presence of genetic variants in TP53 does not provide useful prognostic information for glioblastoma.

## Data Availability

The original contributions presented in the study are included in the article/Supplementary material, further inquiries can be directed to the corresponding author.
