# Supplementary material for: TP53 oncogenic variants as prognostic factors in individuals with glioblastoma: a systematic review and meta-analysis
Source: Front Neurol. 2024 Dec 18;15:1490246. doi: 10.3389/fneur.2024.1490246 (PMC11688405; doi:10.3389/fneur.2024.1490246)

**SUPPLEMENTARY FIGURES**

**Fig S1**. Subgroup analysis for overall survival by gene regions analyzed. The “hotspot exons” subgroup was comprised by reports in which exons 4 or 5 through 8 were evaluated. In the “All exons” subgroup, reports that assessed TP53 in all exons were included. In both subgroups confidence intervals of total effects overlapped the unit. Heterogeneity was partially explained by this analysis, as it decreased in the “All exons” subgroup, however, it increased in the “Hotspot exons” subgroup.


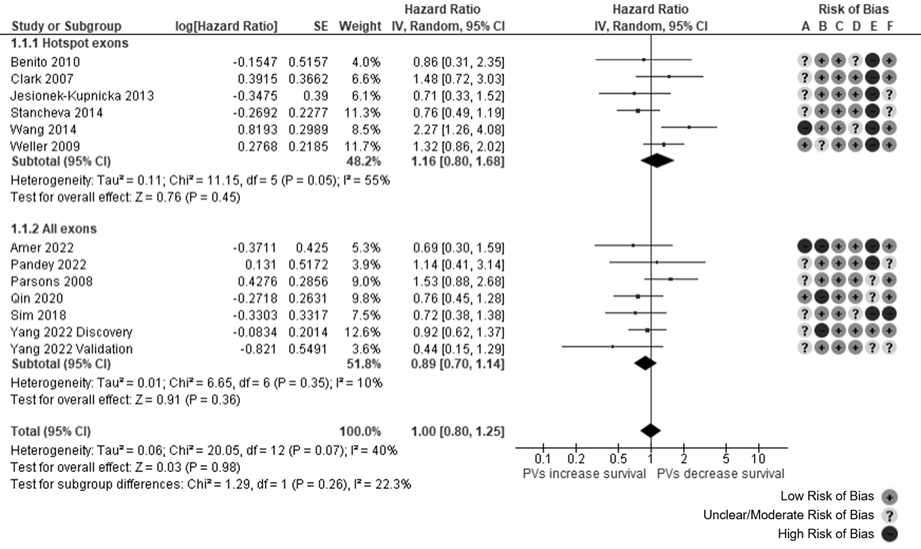


**Fig S2**. Contour-enhanced funnel plot of the meta-analysis addressing overall survival in all patients with glioblastoma. Studies in the white region have a significance level of p>0.10; in the gray, dark gray, and outside area, significance levels are 0.05<p<0.10, 0.01<p<0.05, and p<0.01, respectively.


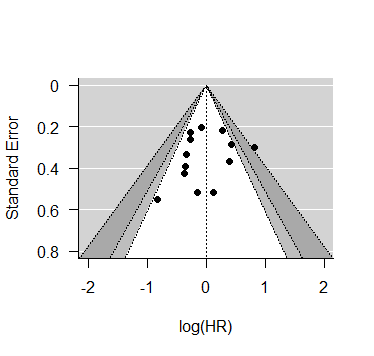


**Fig S3**. Subgroup analysis for overall survival by risk of bias in the QUIPS tool fifth domain, study confounding. Studies were separated in those rated as having low or moderate/unclear risk and those with high risk of bias in the study confounding domain. Total effects did not reach statistical significance in any of the subgroups, and heterogeneity remained virtually unchanged in both.


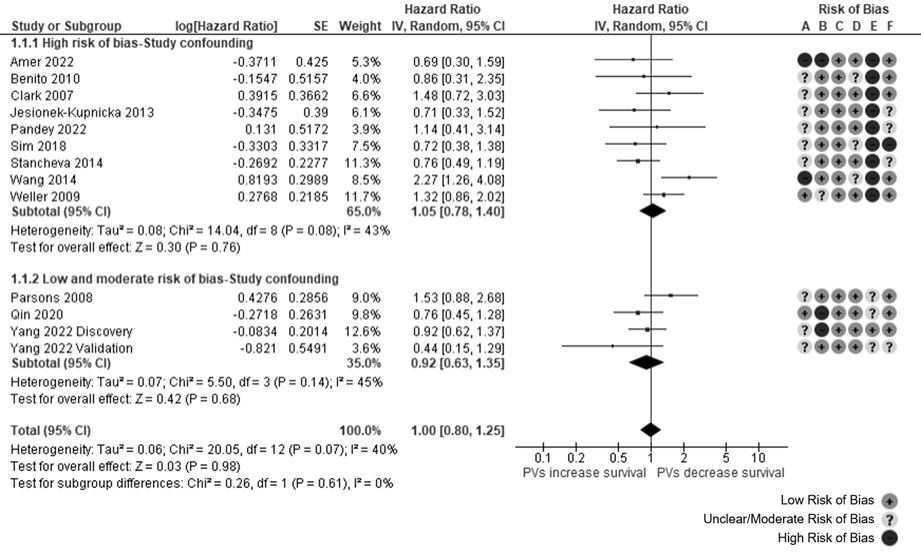


**Fig S4**. Forest plot of the meta-analysis evaluating the impact of TP53 oncogenic variants on OS in individuals with IDH-wildtype glioblastomas, with a summary of the risk of bias assessment through the QUIPS tool. All studies had suboptimal ratings according to the QUIPS tool. Risk of Bias domains: A: Study participation; B: Study attrition; C: Prognostic factor measurement; D: Outcome measurement; E: Study confounding; F: Statistical analysis and reporting. 95% CI: 95% Confidence interval; HR: Hazard ratio; IV: Inverse variance; SE: Standard error.


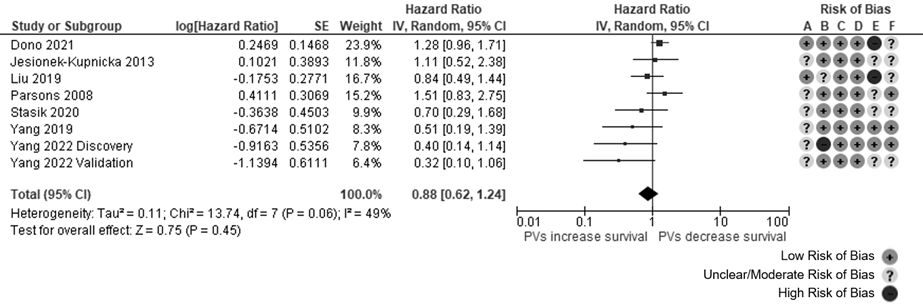

Supplement: Supplementary file 1 [file Table_1.DOCX]
