# Supplementary material for: TP53 oncogenic variants as prognostic factors in individuals with glioblastoma: a systematic review and meta-analysis
Source: Front Neurol. 2024 Dec 18;15:1490246. doi: 10.3389/fneur.2024.1490246 (PMC11688405; doi:10.3389/fneur.2024.1490246)
Supplement: Supplementary file 2 [file Table_2.DOCX]

**Supplementary material 1**

**PubMed search strategy**

(astrocytoma[mh] OR astrocytoma*[tiab] OR astrocytoma*[tw] OR glioma[mh] OR glioma*[tiab] OR glioma*[tw] OR lgg[tiab] OR lgg[tw] OR hgg[tiab] OR hgg[tw] OR glioblastoma[mh] OR glioblastoma*[tiab] OR glioblastoma*[tw] OR gliosarcoma[mh] OR gliosarcoma*[tiab] OR gliosarcoma*[tw] OR brain neoplasms[mh] OR brain neoplasm*[tiab] OR brain neoplasm*[tw] OR brain tum*[tiab] OR brain tum*[tw] OR brain cancer*[tiab] OR brain cancer*[tw] OR glial*[tiab] OR glial*[tw] OR astrocytic[tiab] OR astrocytic[tw] OR glioastrocytoma[tiab] OR glioastrocytoma[tw] OR xanthoastrocytoma[tiab] OR xanthoastrocytoma[tw])

AND (“genes, p53”[mh] OR tp53[tiab] OR tp53[tw] OR p53[tiab] OR p53[tw] OR 17p13[tiab] OR 17p13[tw] OR 17p[tiab] OR 17p[tw])

AND (survival[mh] OR surviv*[tiab] OR surviv*[tw] OR prognosis[tiab] or prognosis[tw] OR prognostic[tiab] OR prognostic[tw] OR os[tiab] OR os[tw] OR pfs[tiab] OR pfs[tw] OR “disease-free survival”[mh] OR dfs[tiab] OR dfs[tw] OR outcome[tiab] OR outcome[tw] OR “disease progression”[mh] OR progress*[tiab] OR progress*[tw] OR recurrence[mh] OR recurren*[tiab] OR recurren*[tw] OR relaps*[tiab] OR relaps*[tw] OR transformation[tiab] OR transformation[tw] OR malignization[tiab] OR malignization[tw])

**Scopus search strategy**

TITLE-ABS-KEY (astrocytoma OR glioma OR lgg OR hgg OR glioblastoma OR gliosarcoma OR ((brain) W/5 (tum* OR cancer OR neoplasm)) OR ((glial OR astrocytic) W/5 (tum* OR cancer OR neoplasm)) OR glioastrocytoma OR xanthoastrocytoma)

AND TITLE-ABS-KEY (tp53 OR p53 OR 17p13 OR 17p)

AND TITLE-ABS-KEY (surviv* OR prognos* OR os OR pfs OR “disease-free survival” OR dfs OR outcome OR “disease progression” OR progress* OR recurren* OR relaps* OR transformation OR malignization)

**Web of Science search strategy**

| #1 | (TS=(astrocytoma* OR glioma* OR lgg* OR hgg* OR glioblastoma* OR gliosarcoma* OR brain NEAR/5 neoplasm* OR brain NEAR/5 tum* OR brain NEAR/5 cancer* OR glial NEAR/5 cancer* OR glial NEAR/5 neoplasm* glial NEAR/5 tum* or astrocytic NEAR/5 cancer OR astrocytic NEAR/5 neoplasm* OR astrocytic NEAR/5 tum* OR glioastrocytoma OR xanthoastrocytoma) OR TI=(astrocytoma* OR glioma* OR lgg* OR hgg* OR glioblastoma* OR gliosarcoma* OR brain NEAR/5 neoplasm* OR brain NEAR/5 tum* OR brain NEAR/5 cancer* OR glial NEAR/5 cancer* OR glial NEAR/5 neoplasm* glial NEAR/5 tum* or astrocytic NEAR/5 cancer OR astrocytic NEAR/5 neoplasm* OR astrocytic NEAR/5 tum* OR glioastrocytoma OR xanthoastrocytoma)  OR AB=(astrocytoma* OR glioma* OR lgg* OR hgg* OR glioblastoma* OR gliosarcoma* OR brain NEAR/5 neoplasm* OR brain NEAR/5 tum* OR brain NEAR/5 cancer* OR glial NEAR/5 cancer* OR glial NEAR/5 neoplasm* glial NEAR/5 tum* or astrocytic NEAR/5 cancer OR astrocytic NEAR/5 neoplasm* OR astrocytic NEAR/5 tum* OR glioastrocytoma OR xanthoastrocytoma))  *Indexes=SCI-EXPANDED, SSCI, A&HCI, CPCI-S, CPCI-SSH, BKCI-S, BKCI-SSH, ESCI Timespan=All years* |
| --- | --- |
| #2 | (TS=(tp53 OR p53 OR 17p13 OR 17p) OR TI=(tp53 OR p53 OR 17p13 OR 17p) OR AB=(tp53 OR p53 OR 17p13 OR 17p))  *Indexes=SCI-EXPANDED, SSCI, A&HCI, CPCI-S, CPCI-SSH, BKCI-S, BKCI-SSH, ESCI Timespan=All years* |
| #3 | (TS=(surviv* OR prognos* OR os OR pfs OR dfs OR “disease free survival” OR “progression free survival” OR outcome* OR progress* OR recurren* OR relaps* OR transformation OR malignization) OR TI=(surviv* OR prognos* OR os OR pfs OR dfs OR “disease free survival” OR “progression free survival” OR outcome* OR progress* OR recurren* OR relaps* OR transformation OR malignization) OR AB=(surviv* OR prognos* OR os OR pfs OR dfs OR “disease free survival” OR “progression free survival” OR outcome* OR progress* OR recurren* OR relaps* OR transformation OR malignization))  *Indexes=SCI-EXPANDED, SSCI, A&HCI, CPCI-S, CPCI-SSH, BKCI-S, BKCI-SSH, ESCI Timespan=All years* |
| #4 | #3 AND #2 AND #1  *Indexes=SCI-EXPANDED, SSCI, A&HCI, CPCI-S, CPCI-SSH, BKCI-S, BKCI-SSH, ESCI Timespan=All years* |

**Biblioteca Virtual en Salud search strategy**

(mh:(“Astrocytoma” OR “Glioblastoma” OR “Glioma” OR “Gliosarcoma” OR “Brain Neoplasms”) OR TW:(glioma$ OR lgg OR hgg OR glioblastoma$ OR gliosarcoma OR brain neoplasm$ OR brain tum$ OR brain cancer$ OR glial OR astrocytic OR glioastrocytoma OR xanthoastrocytoma))

AND

(mh:(“Genes, p53”) OR TW:(tp53 OR p53 OR 17p13 OR 17p))

AND

(mh:(“Survival” OR “Survival Rate” OR “Survival Analysis” OR “Progression-Free Survival” OR “Disease-Free Survival” OR “Disease Progression” OR “Recurrence”) OR TW(survival OR surviv$ OR prognos$ OR os OR pfs OR dfs OR outcome OR recurren$ OR relaps$ OR transformation OR malignization))

**DANS EASY (OpenGrey) search strategy**

Glioblastoma AND TP53
