# Supplementary material for: TP53 oncogenic variants as prognostic factors in individuals with glioblastoma: a systematic review and meta-analysis
Source: Front Neurol. 2024 Dec 18;15:1490246. doi: 10.3389/fneur.2024.1490246 (PMC11688405; doi:10.3389/fneur.2024.1490246)
Supplement: Supplementary file 3 [file Table_3.DOCX]

Approach to quality in Prognosis Studies (QUIPS) for Risk of bias assessment in this review

We assessed the risk of bias across six domains for each prognostic factor-outcome pair. Our general approach was based on modification of the tool proposed by Westby et al 2017.^1^ We used the prompting items from the original QUIPS tool,^2^ together with the signaling questions proposed by McAleenan and colleagues,^3^ to produce a final judgment.

**Domain 1. Study participation**

The prompting items in this domain were intended to guide judgement on the representativity of the study sample in respect to the population of interest.

**Low risk of bias**

The relationship between the prognostic factor and the outcome was unlikely to be different for participating individuals and eligible non-participating individuals. Here we considered participating individuals as the subset of the individuals recruited in the study fulfilling our criteria for target population *and* in whom *TP53* mutational status was determined. We anticipated that, in some of the articles included in the review, the sample of individuals with our characteristics of interest would be a fraction of the total sample included in the study; and the assessment of *TP53* would be only performed in a fraction of said subset. We rated this domain as low risk of bias in a study when all the following conditions were satisfied

- The study sample was consecutively or randomly recruited from the target population. For case control studies, the cases and controls were drawn from the same population;
- Participants were representative of our population of interest:
  - Adults with supratentorial glioblastoma, with an age and sex distribution reasonably similar to the reported in large epidemiological studies;
  - Study follow-up was at least 2 years;
  - Participants did not differ from eligible non-participants and our population of interest for the following variables: sex, age at diagnosis, performance status, treatment received, extent of resection and IDH status;
  - Recruitment rate was high. Given the broad scope of our target population, we considered a recruitment rate as high when 90% or more of adults with glioblastoma in the study setting were included.

**High risk of bias**

The relationship between the prognostic factor and the outcome was very likely to be different for participating individuals and eligible non-participants:

- The sample was selectively recruited. In the case of randomized clinical trials, we assessed whether inclusion and exclusion criteria generated a sample different from our target population. For case and control studies, cases and controls came from different populations;
- Participants were not representative of our target populations:
  - Participants have noticeably differences in terms of sex, age, performance status, extent of resection and IDH status from eligible non-participants and our target population;
  - Study follow-up was inadequate;
  - Recruitment rate was less than 90%.

**Moderate risk of bias**

The relationship between the prognostic factor and the outcome may be different for participating individuals and eligible non-participants, but study reporting did not allow to make a clear decision. Alternatively, we were unsure about the risk of bias secondary to the issue.

**Domain 2. Study attrition**

This domain evaluates whether the study data available adequate represented the study baseline sample

**Low risk of bias**

The relationship between the prognostic factor and the outcome was unlikely to be different for completing and non-completing participants. We considered a study as low risk if any of the following conditions was met:

- No missing outcome data or adequate response rate.
  - A response rate was considered as adequate when outcome data was available for more than 80% of the participating individuals.
- Reasons for missing outcome data were unlikely to be related to the true outcome; or, in time-to-event data, censoring was unlikely to be introducing bias;
- missing outcome data were numerically balanced across *TP53* mutant and *TP53* wild-type groups.

**High risk of bias**

The relationship between the prognostic factor and the outcome was very likely to be different for completing and non-completing participants. A study was declared as presenting high risk of bias should any of the next situations be true:

- deficient response rate (<80%);
- reasons for missing data were likely to be related to the true outcome or *TP53* status, with either imbalance in numbers or reasons for missing data across *TP53* mutant and *TP53* wild-type groups in terms of sex, age, performance status, extent of surgical resection, treatment received or IDH status (when available).

**Moderate risk of bias**

The relationship between the prognostic factor and the outcome may be different for completing and non-completing participants, but study reporting did not allow to make a clear decision. Alternatively, we were unsure about the risk of bias secondary to the issue.

**Domain 3. Prognostic factor measurement**

Domain 3 verified if the prognostic factor of interest was measured appropriately.

**Low risk of bias**

The measurement of the prognostic factor was unlikely to be different for significatively different outcomes or in the presence of different outcome levels. A given study needed to satisfy all the next statements to be declared with low risk of bias:

- an adequate proportion of recruited potential participants (80%) had complete *TP53* status data;
- the method of measurement was adequate and valid;
- mutations in *TP53* were evaluated independently of the outcome;
- mutational status of *TP53* was evaluated with the same method in the same gene regions across all the participants;

**High risk of bias**

The measurement of the prognostic factor was very likely to be different for significatively different outcomes or in the presence of different outcome levels. If any of the following conditions was met, a study received an assignment of high risk of bias

- less than 80% of recruited potential participants had *TP53* mutational status data;
- an unreliable PCR-based method was used to evaluate *TP53* mutational status;
- recruited individuals were selected for *TP53* analysis in function of the outcome (if not in a case cohort study),
- Measurement techniques were different or different *TP53* regions were evaluated;

**Moderate risk of bias**

The relationship between the prognostic factor and the outcome may be different for completing and non-completing participants, but study reporting does not allow to make a clear decision. Alternatively, we were unsure about the risk of bias secondary to the issue.

**Domain 4. Outcome measurement**

In this domain, the quality of the outcome measurement was evaluated.

**Low risk of bias**

The measurement of the outcome was unlikely to be different with respect to different baseline levels of the prognostic factor. We judged a study had a low risk of bias if all the next statements were true:

- a clear definition of the outcome and the follow-up was given;
- the outcome of interest and follow-up was measured similarly for all participant;
- measurement of the outcome was blind to *TP53* mutational status categories.

**High risk of bias**

The measurement of the outcome was very likely to be different related to different baseline levels of the prognostic factor. We gave a study a high risk of bias in any of the following scenarios:

- measurement error varied according to *TP53* status;
- different methods were used for participant with different *TP53* mutational status;
- measurement of the outcome was not blinded.

**Moderate risk of bias**

The relationship between the prognostic factor and the outcome may be different for completing and non-completing participants, but study reporting did not allow to make a clear decision. Alternatively, we were unsure about the risk of bias secondary to the issue.

**Domain 5. Adjustment**

In this domain, it was evaluated if important confounding variables or additional prognostic were appropriately accounted for. This allowed to determine whether the prognostic factor studied was truly related to the change in the outcome

**Low risk of bias**

Important key adjustment factors were appropriately accounted for. We considered a study as presenting low risk of bias only if all the next conditions were true:

- key adjustment factors (i.e., other prognostic factors) were accounted in the study design (*via* matching or stratification), or in the statistical analysis, or adjusted and unadjusted analyses were compared and differences identified;
- key adjustment factors were measured adequately and in the same manner and setting for all the participants;
- measurement of all key factor was valid and reliable;
- treatments have been considered in the analysis if appropriate.

For this study, the following variables were considered key prognostic factors: *IDH1* or *IDH2* mutations, age at diagnosis, sex, extent of resection, chemotherapy, radiotherapy, and performance status at diagnosis.

**High risk of bias**

The observed effect of the prognostic factor on the outcome was very likely to be distorted by another factor related to the prognostic factor and the outcome. If any of the following conditions was met, a study received an assignment of high risk of bias:

- *IDH1* or *IDH2* were not taken into account in either the design or the analysis;
- Adjustment by age was not made;
- Mutations in *IDH1* or *IDH2* were not measured adequately, or they were measured differentially according to the prognostic factor or outcomes;
- interventions were different for different adjustment factors or outcome levels.

**Moderate risk of bias**

The relationship between the prognostic factor and the outcome may be different for completing and non-completing participants, but study reporting did not allow to make a clear decision. Alternatively, we were unsure about the risk of bias secondary to the issue. Key factors other than *IDH1* or *IDH2* were not taken into account.

**Domain 6. Statistical analysis and reporting**

The appropriateness of the statistical analysis performed in the study and the completeness of the reporting was evaluated in this domain.

**Low risk of bias**

The reported results were unlikely to be biased, either in relation to the analysis or the reporting of results. We rated this domain as low risk of bias in a study when all the following conditions were satisfied:

- there was sufficient presentation of the data to assess the adequacy of the analysis;
- the selected statistical model was adequate for the study design;
- the strategy for model building was appropriate;
- there was no selective reporting of the results.

Alternatively:

- Individual participant data was provided.

**High risk of bias**

The reported results were very likely to be biased, either related to the analysis or the reporting of the results. We gave a study a high risk of bias in any of the following scenarios:

- there was insufficient detail to assess the adequacy of the analysis approach;
- the statistical model was inadequate;
- the analysis approach was inadequate;
- results were reported selectively, either on the bases of the findings or statistical significance.

**Moderate risk of bias**

The relationship between the prognostic factor and the outcome may be different for completing and non-completing participants, but study reporting did not allow to make a clear decision. Alternatively, we were unsure about the risk of bias secondary to the issue.
